# Making visible the cost of informal caregivers’ time in Latin America: a case study for major cardiovascular, cancer and respiratory diseases in eight countries

**DOI:** 10.1186/s12889-022-14835-w

**Published:** 2023-01-05

**Authors:** Natalia Espinola, Andrés Pichon-Riviere, Agustín Casarini, Andrea Alcaraz, Ariel Bardach, Caitlin Williams, Federico Rodriguez Cairoli, Federico Augustovski, Alfredo Palacios

**Affiliations:** 1 Institute for Clinical Effectiveness and Health Policy (IECS), C1414CPV Ciudad de Buenos Aires, Argentina; 2 National Council for Scientific and Technical Research (CONICET), Buenos Aires, Argentina; 3 Health Public School, School of Medicine, Buenos Aires University, Buenos Aires, Argentina; 4 Department of Maternal and Child Health, Gillings School of Global Public Health, University of North Carolina at Chapel Hill, Chapel Hill, NC USA; 5 Department of Economics, Buenos Aires University, Buenos Aires, Argentina; 6 Centre for Health Economics (CHE), University of York, York, UK

**Keywords:** Informal care, Indirect costs, Latin American region, Economic evaluation

## Abstract

**Background:**

Informal care is a key element of health care and well-being for society, yet it is scarcely visible and rarely studied in health economic evaluations. This study aims to estimate the time use and cost associated with informal care for cardiovascular diseases, pneumonia and ten different cancers in eight Latin American countries (Argentina, Brazil, Chile, Colombia, Costa Rica, Ecuador, Mexico and Peru).

**Methods:**

We carried out an exhaustive literature review on informal caregivers’ time use, focusing on the selected diseases. We developed a survey for professional caregivers and conducted expert interviews to validate this data in the local context. We used an indirect estimate through the interpolation of the available data, for those cases in which we do not found reliable information. We used the proxy good method to estimate the monetary value of the use of time of informal care. National household surveys databases were processed to obtain the average wage per hour of a proxy of informal caregiver. Estimates were expressed in 2020 US dollars.

**Results:**

The study estimated approximately 1,900 million hours of informal care annually and $ 4,300 million per year in average informal care time cost for these fifteen diseases and eight countries analyzed. Cardiovascular diseases accounted for an informal care burden that ranged from 374 to 555 h per year, while cancers varied from 512 to 1,825 h per year. The informal care time cost share on GDP varied from 0.26% (Mexico) to 1.38% (Brazil), with an average of 0.82% in the studied American countries. Informal care time cost represents between 16 and 44% of the total economic cost (direct medical and informal care cost) associated with health conditions.

**Conclusions:**

The study shows that there is a significant informal care economic burden -frequently overlooked- in different chronic and acute diseases in Latin American countries; and highlights the relevance of including the economic value of informal care in economic evaluations of healthcare.

**Supplementary Information:**

The online version contains supplementary material available at 10.1186/s12889-022-14835-w.

## Background

Informal caregivers are those who care for family members and relatives with short, medium, and long-term diseases; chronic conditions; and/or disabilities without receiving remuneration or compensation [1]. Informal care is an activity with scarce recognition that is strongly feminized and linked to the care economy, making it a contributor to gender inequalities worldwide [2, 3]. International studies show that approximately 70–80% of the people who carry out informal care tasks are women, often the partner of the sick person [4–6].

In Latin America, informal caregivers are the main source of care services for people in a situation of dependency [7, 8]. According to census data from four countries in the region (Brazil, Costa Rica, Ecuador, and Uruguay), only approximately 1% of the population aged 65 and over lives in a residential facility [8]. Yet, the rapid growth of the aging population and the epidemiological transition to chronic and noncommunicable diseases across the region portend an increase in the number of people in a situation of dependency and rising demand for long-term care services [8, 9]. These transitions are occurring against decades-long trends of increasing women's workforce participation and reductions in household size, which impact the time available for women to dedicate to informal care [7, 10]. Women are increasingly expected to work a “double shift” of paid and unpaid work, creating unsustainable and unhealthy situations of stress, overload, and burnout that deepen gender inequity [11–13]. Although some countries in Latin America have made proposals to advance public policy that supports informal caregivers, progress has thus far been limited [7].

The distribution of tasks related to the care economy is influenced by the gendered division of work, which itself arises from social and cultural stereotypes regarding the societally prescribed roles of women and men. Accordingly, a higher burden of unpaid informal care falls on women [14]. It is estimated that around 90% of women in Latin American countries participate in unpaid care and housework tasks, spending twice as much time on these tasks as men [14]. When men do participate in unpaid caregiving, they often substitute the time they would otherwise spend on household chores, leaving the time set aside for paid work and leisure activities untouched. In contrast, women take on unpaid caregiving alongside other unpaid household chores, reducing the time available for paid work and leisure [15, 16]. This unfair distribution of informal care work negatively affects women's educational, social, and professional decisions, as well as their performance and aspirations in the labor market, widening gender inequalities [17].

Even when tasks within the care economy are more evenly distributed, emergencies (such as the COVID-19 pandemic) can cause households to relapse into more gender-proscriptive roles, with women expected to pick up the slack around increased unexpected care work [3, 18]. Data suggest that in two-earner heterosexual households, gendered discrepancies in earnings, combined with social norms, often nudge women out of the workforce faster than men when new caregiving responsibilities arise [19–22]. In short, the conditions of paid work are closely linked to how unpaid tasks are distributed.

The assumption that caregiving is women’s responsibility limits the recognition of caregiving as labor, precluding it from consideration within economic evaluations. For example, the time survey used in Latin American countries to characterize how people of different ages distribute their time across to the activities they carry out inside and outside the home does not report on informal care for sick people [10]. Economic models typically do not recognize care work as a generator of value, leading to an undervaluation of informal caregiving [23]. Yet those economic models that do take care work into account suggest that informal caregiving represents a substantial fraction of the total economy. In high-income countries, the value of informal care as a percentage of GDP ranges from 0.3% (France) to 7.4% (United Kingdom) [24–27]. These values may be even higher in low- and middle-income countries. For example, according to an estimate made in Argentina, unpaid care and housework was equivalent to 15.9% of Gross Domestic Product (GDP) in 2020 [28].

Further, though patients are not isolated individuals, economic evaluations of health and healthcare programs and/or policies often ignore the costs and health outcomes of caregivers. Krol et al. performed a systematic review to explore the inclusion of informal care in economic evaluations and the potential impact of the costs and effects of informal caregiving on cost-effectiveness outcomes [29]. Out of 100 economic evaluations, only 23 included the costs and/or effects of informal caregiving. In 8 studies, the impact of including or excluding informal care costs or effects on cost-effectiveness outcomes was significant. Thus, economic evaluations that overlook informal caregiving may underestimate total social costs, especially in those diseases where informal care provides a large part of the total care or where the relative impact is high. Economic evaluations tightly focused on health care costs should at least include caregivers’ health outcomes, since they constitute part of the health burden associated with the event or situation analyzed [30–32]. Ignoring the impact of informal care provision results in poorly informed decisions and little awareness of their distributional consequences.

The purpose of the study is to estimate the economic value of time spent on the informal care associated with fifteen major non-communicable diseases (five cardiovascular diseases and ten different cancers) and pneumonia in eight Latin American countries: Argentina, Brazil, Chile, Colombia, Costa Rica, Ecuador, Mexico, and Peru. These countries lead the economic income of the region and represent 80% of the Latin American population. This information will help quantify the extent of the current problem regarding informal care and can be used to develop equity-informed public health policies.

## Methods

### Care time estimation

As mentioned above, there are microdata on the use of time in unpaid care tasks in the Latin American region. However, these data do not present the information needed for this study: the number of hours spent on informal care by disease. Therefore, we decided to collect this information through a literature review.

We carried out an exhaustive literature review on costs and use of time of informal caregivers. The literature review was conducted in PubMed and LILACS until June 2020, with no language restrictions. The search strategy was limited to systematic reviews (SRs) that contained information on costs and time use of informal caregivers, focusing on selected diseases: cardiovascular disease, chronic obstructive pulmonary disease and ten cancers (lung, mouth, esophageal, stomach, pancreatic, kidney, laryngeal, leukemia, bladder, neck). The search strategies are presented in the Supplementary Material.

Few systematic reviews presented information on informal care hours required due to illness. Then, we screened the full text of each of the primary studies in the SR for the required data. The majority of the studies retrieved were from European countries and the United States, although some studies were from Chile, Brazil, and Mexico.

In summary, only three SRs and three primary studies of SR presented information on daily hours spent on informal care by disease [14, 33–38]. These studies showed that the hours spent on informal care for stroke ranged from 6 to 11 h per day. One SR and two primary studies reported data on daily hours of care for the cancers analyzed. The hours of informal care for cancer ranged from 7 to 11 h per day [39–41]. For pulmonary diseases, two primary studies about chronic obstructive pulmonary disease (COPD) found that the informal caregiver spends approximately between 2–12 h per day depending on its severity [4, 42]. Two Latin American articles reported the data with results like those mentioned above in cancer and stroke [14, 43].

Additionally, we conducted a survey with seven formal caregivers from Argentina to validate the data in the local context. We sent them a self-administered questionnaire to validate the data on the number of daily hours of care according to illness, obtained from the literature review. Next, we interviewed clinical specialists (cardiologists, oncologists, and pulmonologists) to validate our preliminary estimates. From this phase, other determinants emerged for a final adjustment of the values, such as the rate of use of care and the number of days of care per year, which depend on the severity of the disease and on whether the condition is chronic or acute (See Supplementary Table S1).

Finally, we carried out an indirect estimate through interpolation of available data, for those cases (mainly cancers) where we did not find data in the literature and for which there was no clear validation (*n* = 9). We performed a simple interpolation using a linear fit through econometric estimation.^1^ We estimated the linear relationship between the hours per day of informal care for the health events considered (*n* = 10) and the quality of life (utilities) of the patients in these health events, based on empirical evidence [44, 45]. To do this, we used the method of ordinary least squares. It is the most common estimation method when fitting a linear regression model on the parameters. This method has many advantages in terms of its ease of use and the suitability of the mathematical statistical approach that allows it to adapt to the assumptions for the econometric models and, it allows finding the Best Linear Unbiased Estimators [46].^2^ The adjusted R-squared of the regression was 0.4106. With the results of the regression coefficients, missing data were imputed. Utilities were extracted from a literature review (See Supplementary Table S1).

### Informal caregiver shadow price

To estimate the economic value of informal care, several methods have been discussed in the literature and have been applied in previous research [17]. The most commonly used methods are the opportunity cost method and proxy good method [35]. With the first method, informal care hours are valued based on the use of time in alternative tasks lost, such as paid work. With the second method, the value of the time spent in informal care is determined by the market (labor) prices of a ‘close market substitute’. Such estimation requires the availability of a market substitute for the non-market goods, which is assumed to be near perfect (e.g., home care nursing) [29]. In our data set, we did not have information on the caregiver’s primary occupation and so could not calculate the value of alternative time use. Thus, in this study, we used the proxy good method.

Then, following the proxy good method, we assumed the average hourly wage of those who work in health-related social assistance as a proxy for the shadow price of informal caregivers. This information was estimated from microdata of nationally representative household surveys, provided by the national statistics office of each of the countries included in this study [47–54]. These surveys provide socioeconomic and labor characteristics of individuals, and due to their two stages probabilistic and stratified sampling method, permit the elaboration of nationally representative statistics. This information is shown in the Supplementary Table S2. A strong heterogeneity is observed in the monetary values per hour/day of informal care between countries. It is estimated that Chile ($ 4.12) has the relatively highest monetary value of one hour of informal caregiver, and Mexico ($ 1.05) has the relatively lowest monetary value.

With the data of the estimated hours of informal care per day that a health condition requires and the hourly wage, we obtained the daily cost of the average informal caregiver per health condition. By multiplying the annualized cost by the total number of cases of the health condition in a country in a year, we obtained estimates of the annual economic burden of informal care associated with each of the specified health conditions for the country. The 95% confidence interval values were estimated using the standard error of the results of the econometric equation.

We estimated the costs in local currency units. In the cases where the available survey data was collected prior to 2020, we adjusted data to 2020 using the Consumer Price Index (CPI). Then, we converted to 2020 US dollars (USD) using the average exchange rates for each local currency, which were obtained from the web page of each Central Bank [55–61]. The Gross Domestic Product (GDP) was extracted from databases of multilateral organizations [62, 63]. This information is shown in the Supplementary Table S2.

### Estimation of burden diseases and medical direct costs

Data on the total number of cases per disease and the total medical direct costs in adults by health condition and country were extracted from an update of the work by Pichon-Riviere et al. (personal communication) [64]. This study used a model based on a first-order Monte Carlo micro-simulation model, which assesses the burden of disease and the economic burden of tobacco consumption, and estimates the impact of different tobacco control interventions. The study estimates the number of total cases and the direct medical costs of each health condition considered. The research presented here is part of a study based on this initial investigation, and in which the cases and costs were updated to the year 2020. Supplementary Table S3 shows the number of cases and Supplementary Table S4 shows the total medical direct costs, per health condition and country in a year (both people who are in their first year and in subsequent years of the disease).

## Results

Table 1 shows the average hours per year spent on informal care for each health condition. For heart conditions, time spent ranged between 183 and 374 h/year, while for stroke time spent was estimated to be 555 h/year in the first year and 379 h/year in subsequent years. For COPD, the hours of informal care required ranged from 0 to 2086 annually, according to severity. For cancers, it is estimated that the hours of informal care required per day range from 512 h/year (for a patient with leukemia) to 1825 h/year (in a patient with stomach or pancreatic cancer). For pneumonia, an acute disease, an estimated 131 h/year of informal care are required.

**Table 1:** Mean hours/year of informal care required by health conditions

Health condition	Mean hours/year	IC 95%	
		**IL**	**UL**
Acute Myocardial Infarction (first year)	374	321	427
Coronary event no AMI (first year)	351	299	405
Coronary event (care post-event)	183	128	237
Stroke (first year)	555	500	606
Stroke (year 2 +)	379	325	431
Pneumonia	131	77	183
Mild COPD^a^	0	0	55
Moderate COPD^a^	250	197	303
Severe COPD^a^	2,086	2,033	2,139
Lung cancer	1,290	1,237	1,343
Mouth cancer	877	825	931
Esophageal cancer	1,436	1,383	1,489
Stomach cancer	1,825	1,770	1,876
Pancreatic cancer	1,825	1,770	1,876
Kidney cancer	706	653	759
Laryngeal cancer	804	752	858
Leukemia	512	460	566
Bladder cancer	1,202	1,150	1,256
Neck cancer	813	759	865

^a^*COPD* Chronic obstructive pulmonary disease

The monetary values of informal care time vary based on the number of hours per day spent per health condition as well as the wage proxy of informal caregivers by country. For example, the cost of providing informal care for a patient with lung cancer varied between $ 3.70 (IC 95%, 3.56–6.16) per day in Mexico to $ 14.57 (IC 95%, 13.670–15.50) per day in Chile. On average, an informal caregiver is estimated to have a monetary value of his or her time of $9 for caring for a patient with lung cancer, in the Latin American countries included in this study (See Supplementary Table S5).

Furthermore, in Table 2 we estimated the annual total of informal care time cost using the cost per year of informal care time and the total number of cases annually per health condition in each country. The estimated annual total cost of informal care time varied between $ 362 million (Costa Rica) and $ 17,564 million (Brazil) for the selected health conditions. Per million inhabitants, the total cost of the time required for informal caregivers varied between $ 53 million (Mexico) and $ 298 million (Chile) annually. Cardiovascular diseases, stroke, and COPD together account for 80–90% of the total cost of informal care time by country. Among cancers, the greatest economic burden from informal care time was attributable to lung, stomach and pancreatic cancer, which accounted for 5–11% of the total cost of informal caregiving by country.

**Table 2:** Annual total cost of informal care time by health condition and country, in million 2020 dollars

Events	Argentina		Brazil		Chile		Colombia	
	Mean	IC 95%	Mean	IC 95%	Mean	IC 95%	Mean	IC 95%
**Coronary events**	1,206.51	[960.59–1494.79]	9,978.66	[7002.05–14,206.1]	935.49	[742.15–1166.83]	630.11	[477.81–832.7]
**Stroke**	707.36	[616.04–807.23]	3,130.90	[2384.63–4276.21]	987.86	[856.93–1132.09]	866.17	[698.89–1087.83]
**Pneumonia**	55.70	[33.43–90.93]	268.89	[153.33–474.47]	15.69	[9.44–25.36]	10.07	[5.85–16.94]
**COPD**	567.33	[509.02–642.47]	2,548.49	[1910.13–3516.07]	587.03	[518.7–672.79]	307.69	[246.62–388.59]
**Lung cancer**	102.83	[98.11–107.26]	300.94	[232.04–401.3]	67.44	[63.29–71.77]	27.54	[22.94–33.16]
**Mouth cancer**	19.72	[18.6–20.83]	142.54	[110.41–189.67]	9.55	[8.88–10.25]	7.80	[6.48–9.44]
**Esophageal cancer**	31.54	[30.18–32.87]	158.32	[122.1–208.94]	20.66	[19.41–22.03]	6.91	[5.81–8.32]
**Stomach cancer**	69.07	[66.81–71.4]	360.96	[278.67–475.97]	144.91	[136.79–153.04]	68.71	[57.35–82.65]
**Pancreatics cancer**	89.41	[86.65–92.27]	250.86	[194.25–330.21]	58.69	[55.6–61.95]	25.47	[21.26–30.56]
**Kidney cancer**	29.60	[27.59–31.94]	54.90	[42.59–72.37]	24.38	[22.44–26.78]	4.84	[3.98–5.96]
**Laryngeal cancer**	11.93	[11.11–12.77]	70.96	[54.98–94.28]	4.48	[4.13–4.87]	3.57	[2.97–4.37]
**Leukemia**	12.44	[874–2047]	47.33	[1577-404]	9.97	[703–1301]	6.22	[431–789]
**Bladder cancer**	42.02	[2999–6767]	142.80	[4755–12039]	27.62	[1979–3643]	9.48	[679–1188]
**Neck cancer**	28.70	[2046–4636]	109.07	[3686–9376]	18.30	[1306–2415]	14.10	[1007-177]
**Total**	**$2974**		**$17,566**		**$2,912**		**$1,989**	
**Total per million inhabitants**	**$145,763,411**		**$178,995,457**		**$298,011,466**		**$90,486,014**	

Events	Costa Rica		Ecuador		Mexico		Peru	
	Mean	IC 95%	Mean	IC 95%	Mean	IC 95%	Mean	IC 95%
**Coronary events**	220.30	[175.24–274.43]	430.81	[199.72–895.89]	1,133.99	[908.82–1406.62]	168.23	[76.43–339.72]
**Stroke**	43.38	[38.08–49.5]	355.88	[161.44–740.31]	901.44	[794.6–1027.03]	459.42	[212.99–919.51]
**Pneumonia**	2.74	[1.68–4.4]	13.36	[5.75–31.49]	36.38	[23.25–59.09]	34.67	[14.54–79.57]
**COPD**	52.37	[47.12–59.11]	160.78	[75.02–333.08]	482.16	[433.79–548.29]	449.68	[214.07–902.86]
**Lung cancer**	5.43	[5.2–5.63]	10.99	[5.15–21.97]	33.35	[32.09–89.14]	28.04	[13.63–54.82]
**Mouth cancer**	1.82	[1.72–1.92]	3.05	[1.4–6.09]	10.43	[9.87–27.91]	9.10	[4.36–18.16]
**Esophageal cancer**	1.55	[1.5–1.61]	3.18	[1.49–6.44]	7.21	[6.97–19.16]	5.75	[2.81–11.42]
**Stomach cancer**	18.78	[18.28–19.27]	46.44	[21.91–93.64]	55.29	[53.77–147.05]	93.26	[45.23–185.35]
**Pancreatics cancer**	6.30	[6.14–6.47]	11.76	[5.5–23.84]	37.60	[36.56–100.07]	28.69	[13.96–56.58]
**Kidney cancer**	1.46	[1.36–1.57]	3.00	[1.38–6.24]	12.33	[11.5–32.48]	9.33	[4.45–18.35]
**Laryngeal cancer**	1.06	[0.99–1.13]	1.35	[0.63–2.78]	4.87	[4.55–12.75]	2.00	[0.93–3.94]
**Leukemia**	1.46	[123–168]	4.15	[342-49]	8.21	[274–2175]	7.76	[645–1022]
**Bladder cancer**	2.92	[255–332]	4.61	[388–541]	9.87	[326–2604]	9.52	[804–1233]
**Neck cancer**	2.69	[231–308]	11.83	[991–1382]	22.12	[711–5974]	27.02	[2275–3532]
**Total**	**$362**		**$1,061**		**$2,755**		**$1,332**	
**Total per million inhabitants**	**$157,517,703**		**$167,508,373**		**$53,193,394**		**$99,509,016**	

**Fig. 1:**
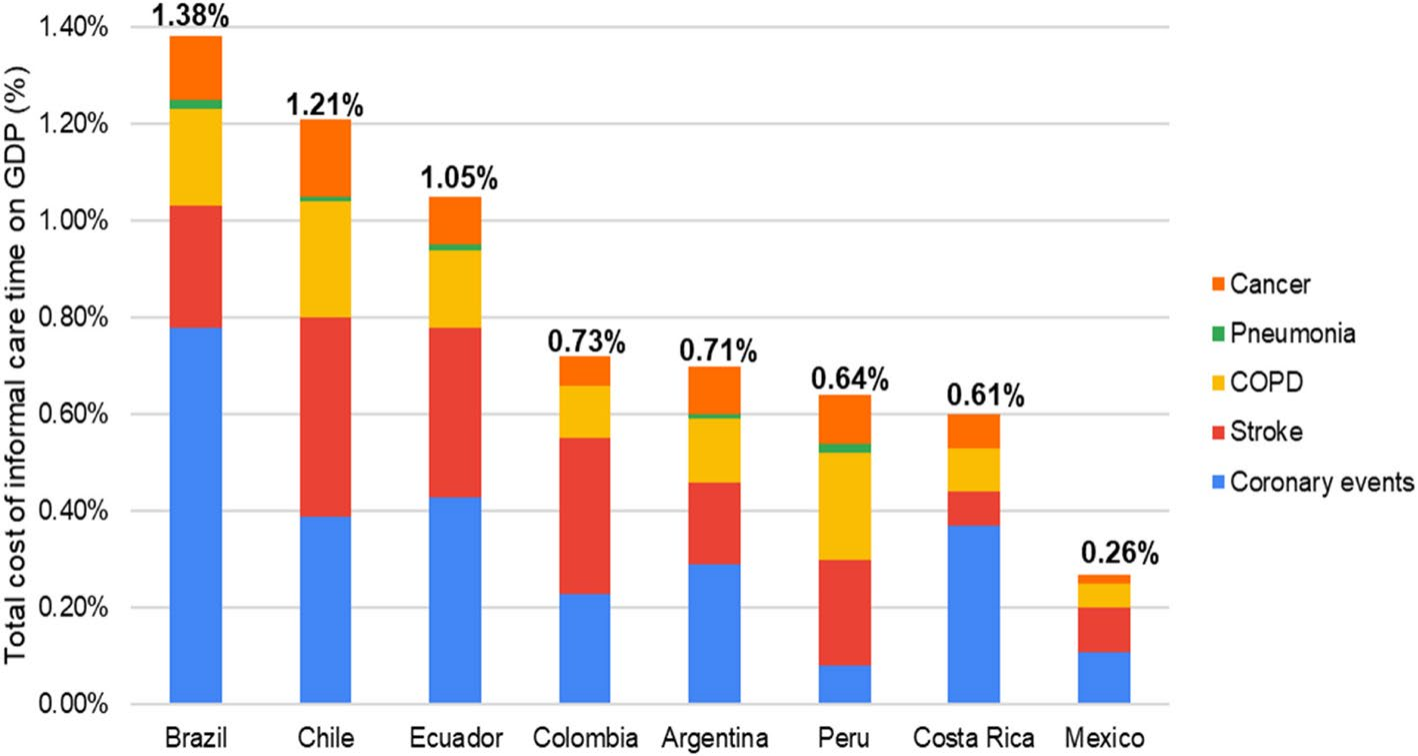
Informal care economic burden as a proportion of GDP by disease and country

**Fig. 2:**
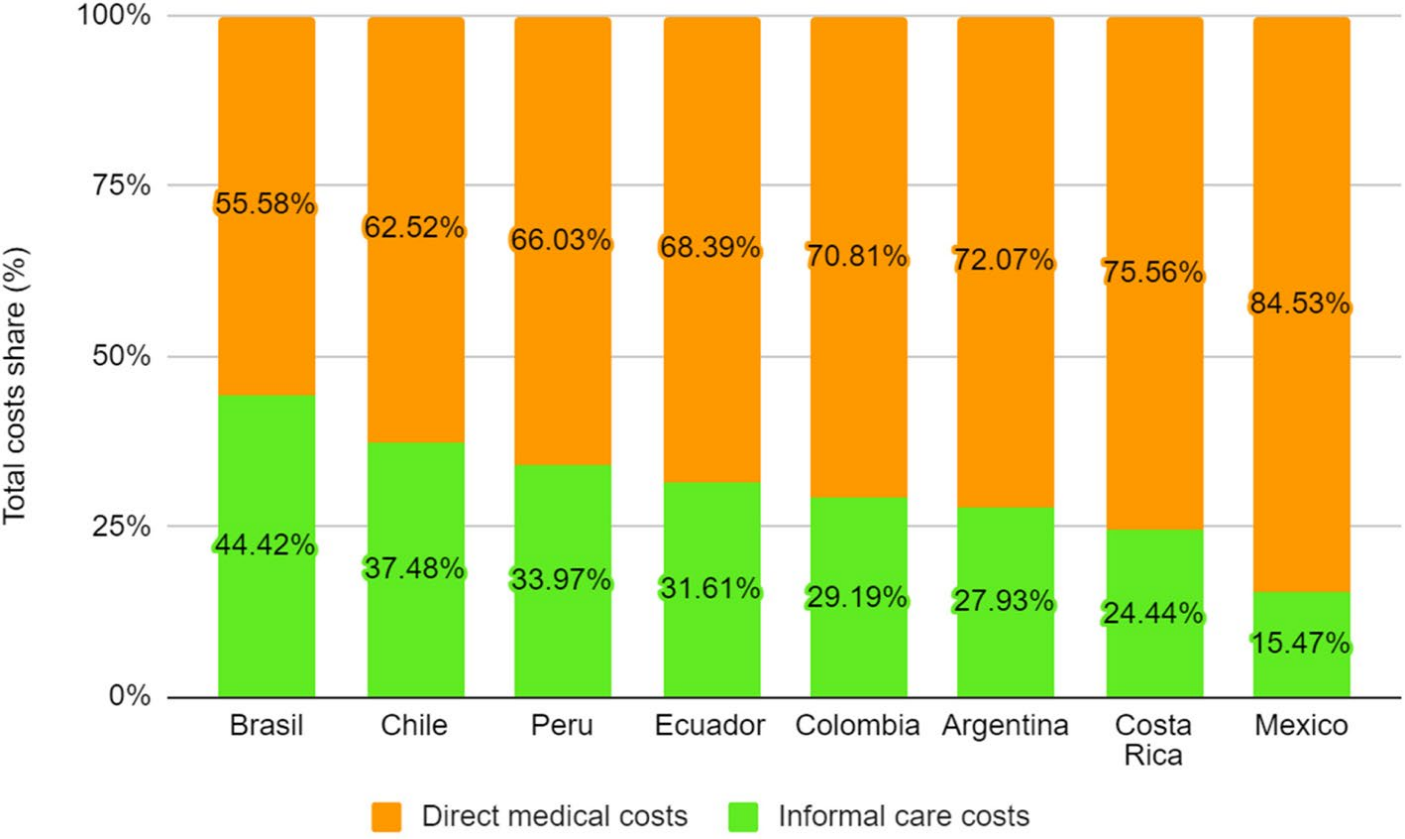
Share (%) of informal care cost and direct medical cost in total cost by country

Finally, Fig. 1 shows the annual total cost of informal care time in relation to GDP of each country. This economic burden varied between 1.38% in Brazil to 0.26% in Mexico, representing an average of 0.82% of GDP. As we mentioned earlier, the greatest contribution comes from cardiovascular diseases, stroke, and COPD. In addition, Fig. 2 presents the contribution of the informal care cost and direct medical cost in the annual total cost for the selected health conditions. The cost of informal care varies between 16% (Mexico) and 44% (Brazil).

## Discussion

Our study found that informal care poses a significant economic burden in Latin American countries, accounting for an average of approximately 1.9 billion hours annually with a monetary equivalent of $ 3.9 billion per year for these fifteen major cardiovascular, cancer, and respiratory diseases in the eight countries analyzed in 2020. The estimated value of informal care time in each country varies according to the most prevalent diseases, total number of cases, and hourly wages. Among the diseases analyzed, cardiovascular diseases, stroke, and COPD account for 80–90% of the burden of informal care. In our study, the total estimated cost of informal care ranges from 0.26 to 1.38% of annual GDP, in line with estimates in the literature from high-income countries [24–27]. No estimates were found for Latin American countries in the reviewed literature. Country-level variance in estimates has been attributed to differences in estimation methodology, as well as demographics, cultural attitudes toward caring, health policy, and total GDP [24–27]. In addition, our findings suggest that informal care time represents between 16% (Mexico) and 44% (Brazil) of the total cost associated with the studied diseases (direct medical cost and informal care cost). This highlights the importance of including the cost of informal care time in analyses of the economic burden of a disease, in order to quantify the true social, health, and economic costs – costs set to grow with the epidemiologic and demographic transitions the regions is experiencing.

Gender-disaggregated data are critical for characterizing the distribution of those costs. Yet gender of caregivers was not reported in the time use data literature used to create the estimates in our study, limiting our ability to determine the gender-differential impact of caregiving in the studied countries. However, other studies in Latin America document the greater presence of women in care tasks and domestic work [3, 23]. In most of the studies analyzed, the caregiver is a woman (70–80% of informal caregivers), aged 50–60, and the wife of the patient [4, 34, 38]. Thus, a substantial portion of the burden we estimate is likely borne by women. These findings underscore the need for greater access to gender-disaggregated data and gender-sensitive analyses [65, 66]. They also point to the need for gender-responsive and gender-transformative policies with respect to aging and informal care.

With respect to gender, policies can run the gamut from gender unequal (those that further entrench gender inequities) to gender transformative (those that seek to transform the underlying harmful gender roles and norms that produce gender inequity) [67]. Current Latin American policies that seek to offset the burden of caregiving largely sit in the middle of this continuum and ignore gender [7]. For example, Uruguay (the first Latin American country to set up a national system to provide assistance to care-dependent people) offers services like personal home care assistants for 80 h per month for severely care-dependent people over age 80; telecare for people over age 70 who are moderately or mildly care-dependent; and services at free day centers for moderately or mildly care-dependent people over age 65 [7]. The “Cuida Chile” Program in Chile provides personal home care assistance to adults who are severely or moderately care-dependent and live in low-income households [7]. In Argentina, the PAMI program covers health and social services, including long-term care [7]. These innovative programs offer needed services to care-dependent people, but they overlook caregivers and thus may inadvertently privilege men to receive state assistance over women (at the same time, greater life expectancy among women may mean that more women benefit from these programs than men, even as the burden of caregiving falls disproportionately on women). Further, these programs only address the costs associated with formal care and thus ignore the needs of the feminized informal caregiver class. By quantifying the cost of informal caregiving, we hope to draw attention to this largely invisibilized issue and prompt the development of policy informed by a gender perspective [68].

This study has several limitations. First, we assume that the care time by health condition is the same for all countries, which may not be true considering the demographic, cultural, and public policy differences between the countries. Future studies could apply interview and survey techniques to professional caregivers (as a proxy) in each country to adapt estimates for context. Alternatively, national time use surveys conducted in Latin American countries could be adapted to capture data on the allocation of time to caregiving due to disease, allowing for more detailed analyses.

Second, although we used wage rates, it is recognized that assuming wage as a measure of the good proxy of the use time cost is not entirely correct. When using the wage rates of, for example, health professionals (in our case, health care related social assistance) as a proxy value, formal care and informal care are assumed to be perfect substitutes. For example, it is not assumed that there are differences in efficiency and quality. It is also assumed that informal care does not imply direct (dis)utility. This means that neither the care recipient nor the informal caregiver enjoy the fact that the latter provides the care [17]. Also, the lost wages of a worker can be an over/underestimation of the value of their time. Despite all this, we consider that health care related social assistance salary continues to be a good and practical approximation to the true shadow value.

Third, due to the lack of detailed time use information, we were unable to use the opportunity cost method and present estimates from two different methods for comparison. Empiric evidence suggests that estimates made using the proxy good method are higher than those obtained using the opportunity cost method (approximately 45% higher values) and present greater variability [35]. Therefore, our estimates may overestimate the economic burden of informal care. However, there is no conclusive evidence on which of the two methods is better [17].

Despite these limitations, this study provides useful evidence that quantifies the magnitude of the challenge facing health policymakers in Latin America. Further, it highlights the need to generate more detailed national statistics on informal caregivers in the region, in order to learn more about the population’s socioeconomic characteristics, unmet needs, quality of life, and other considerations. Such improved statistics will allow for more detailed analyzes, improve estimates of the economic burden of informal care, and inform gender-responsive and gender-transformative policies that can reshape the face of care in the region.

## Conclusion

The study provides an estimate of the time spent on, and economic value associated with, informal caregiving in Latin America. It helps to make visible the burden informal care represents for caregivers and society at large, as well as showcasing the heterogeneity of needs associated with different diseases across different countries. The study highlights the relevance of including the economic value of informal care in the economic evaluations of healthcare. In addition, it provides evidence to support calls for ensuring a gender perspective informs the development of long-term care policies.

## Supplementary Information

**Additional file 1:** SM. Search strategies in the literature review. **Table S1.** Inputs used to estimate the hours per day of informal care by events. **Table S2.** Hourly wage used to proxy of the market value of informal care time, rate exchange and GDP by country, 2020 USD. **Table S3.** Total number of cases per health events and country in a year. **Table S4.** Medical direct costs per health event and country, million 2020 USD. **Table S5**. Average cost per day of informal care by health condition and country, in 2020 dollars. 

## Data Availability

The datasets generated and analysed during the current study are presented in the manuscript and publicly available. The survey access links can be found in the references.

## Footnotes

1 We tried other forms of relationship such as logarithmic, and the results were very similar.

2 We carry out all the tests to evaluate the assumptions of an ordinary least squares model. The reader can request them from the authors.

## References

[1] Koopmanschap MA, van Exel JNA, van den Berg B, Brouwer WBF (2008). An overview of methods and applications to value informal care in economic evaluations of healthcare. Pharmacoeconomics.

[2] García Calvente M, del Río LM, Marcos Marcos J (2011). Desigualdades de género en el deterioro de la salud como consecuencia del cuidado informal en España. Gac Sanit.

[3] CEPAL. Cuidados y mujeres en tiempos de COVID‐19: la experiencia en la Argentina. In: Comisión Económica para América Latina y el Caribe. 2020. https://repositorio.cepal.org/bitstream/handle/11362/46453/1/S2000784_es.pdf. Accessed Jul 2021.

[4] Miravitlles M, Peña-Longobardo LM, Oliva-Moreno J, Hidalgo-Vega Á (2015). Caregivers’ burden in patients with COPD. Int J Chron Obstruct Pulmon Dis.

[5] Rabier H, Serrier H, Schott A-M, Mewton N, Ovize M, Nighoghossian N, Duclos A, Colin C (2019). Economic valuation of informal care provided to people after a myocardial infarction in France. BMC Health Serv Res.

[6] Ochoa CY, Buchanan Lunsford N, Lee Smith J (2020). Impact of informal cancer caregiving across the cancer experience: a systematic literature review of quality of life. Palliat Support Care.

[7] Cafagna G, Aranco N, Ibarrarán P, Medellín N, Oliveri ML, Stampini M. Envejecer con cuidado: Atención a la dependencia en América Latina y el Caribe. 2019. 10.18235/0001972

[8] Álvarez F, Toledo M, Allub L, Alves G, de la Mata D, Estrada R, Daude C, Others. Pension and healthcare systems in Latin America: Challenges posed by aging, technological change, and informality. Report on Economic Development, CAF Development Bank Of Latinamerica, number 1730, September 2021. https://ideas.repec.org/b/dbl/dblrep/1730.html.

[9] Santosa A, Wall S, Fottrell E, Högberg U, Byass P (2014). The development and experience of epidemiological transition theory over four decades: a systematic review. Glob Health Action.

[10] Pedrero NM (2008). Propuesta metodológica para medir y valorar el cuidado de la salud doméstico no remunerado. Organización Panamericana de la Salud (ed) La economía invisible y las desigualdades de género. La importancia de medir y valorar el trabajo no remunerado.

[11] Phillips D, Paul G, Fahy M, Dowling-Hetherington L, Kroll T, Moloney B, Duffy C, Fealy G, Lafferty A (2020). The invisible workforce during the COVID-19 pandemic: family carers at the frontline. HRB Open Res.

[12] Heggeness ML (2020). Estimating the immediate impact of the COVID-19 shock on parental attachment to the labor market and the double bind of mothers. Rev Econ Househ.

[13] Raj A. Gender and the COVID-19 pandemic: Multinational research indicate that we must support and compensate LMIC women’s leadership in crises. eClinical Med. 2022;53:101748 10.1016/j.eclinm.2022.101748 PMC9671551 36415747

[14] Organización Panamericana de la Salud. La economía invisible y las desigualdades de género: La importancia de medir y valorar el trabajo no remunerado. In: Organización Panamericana de la Salud. 2008. https://iris.paho.org/handle/10665.2/6034. Accessed Jul 2021.

[15] Miller R, Sedai AK. Opportunity costs of unpaid caregiving: evidence from panel time diaries. CAMA Work Pap. 2021. 10.2139/ssrn.3827577.

[16] Stanfors M, Jacobs JC, Neilson J (2019). Caregiving time costs and trade-offs: gender differences in Sweden, the UK, and Canada. SSM Popul Health.

[17] van den Berg B, Brouwer WBF, Koopmanschap MA (2004). Economic valuation of informal care. an overview of methods and applications. Eur J Health Econ.

[18] Collins C, Landivar LC, Ruppanner L, Scarborough WJ (2021). COVID-19 and the gender gap in work hours. Gend Work Organ.

[19] Lu Y, Wang JS-H, Han W-J (2017). Women’s short-term employment trajectories following birth: patterns, determinants, and variations by race/ethnicity and nativity. Demography.

[20] Barnett RC. Dual-Earner Couples: Good/Bad for Her And/or Him? In From Work-Family Balance to Work-Family Interaction, edited by Diane F. Halpern, 278:151–71. Lawrence Erlbaum Associates Publishers, xix: Mahwah, NJ, US. ISBN: 9781410612090

[21] Weeden KA, Cha Y, Bucca M (2016). Long work hours, part-time work, and trends in the gender gap in pay, the motherhood wage penalty, and the fatherhood wage premium. RSF Russell Sage Found J Soc Sci.

[22] Webber G, Williams C (2008). Part-time work and the gender division of labor. Qual Sociol.

[23] Comisión Económica para América Latina (CEPAL). La economía del cuidado como acelerador del cambio estructural con igualdad. In: Observatorio de Igualdad de Género de América Latina y el Caribe. 2020. https://oig.cepal.org/sites/default/files/no30_esp_-_economia_del_cuidado.pdf. Accessed July 2021.

[24] Oliva-Moreno J, Peña-Longobardo LM, Vilaplana-Prieto C (2015). An estimation of the value of informal care provided to dependent people in Spain. Appl Health Econ Health Policy.

[25] Buckner L, Yeandle S. Valuing carers 2015. The rising value of carers’ support. In: Carers UK. 2021. https://socialcare.wales/cms-assets/documents/hub-downloads/Valuing_Carers_2015.pdf.

[26] Deloitte Access Economics. The economic value of informal care in Australia in 2015. In: Delloite. 2015. https://www2.deloitte.com/au/en/pages/economics/articles/economic-value-informal-care-Australia-2015.html. Accessed Nov 2021.

[27] Deloitte Access Economics. The value of informal care in 2020. Caring comes at a cost. In: Deloitte. 2020. https://www2.deloitte.com/au/en/pages/economics/articles/value-of-informal-care-2020.html. Accessed Nov 2021.

[28] Dirección Nacional de Economía, Igualdad y Género (2020). Los cuidados, un sector económico estratégico Medición del aporte del Trabajo Doméstico y de Cuidados no Remunerado al Producto Interno Bruto.

[29] Krol M, Papenburg J, van Exel J (2015). Does including informal care in economic evaluations matter? A systematic review of inclusion and impact of informal care in cost-effectiveness studies. Pharmacoeconomics.

[30] Girgis A, Lambert S, Johnson C, Waller A, Currow D (2013). Physical, psychosocial, relationship, and economic burden of caring for people with cancer: a review. J Oncol Pract.

[31] Girgis A, Lambert S. Cost of informal caregiving in cancer care. Cancer Forum. 2017;41:1–7.

[32] Cohen SA, Kunicki ZJ, Drohan MM, Greaney ML (2021). Exploring changes in caregiver burden and caregiving intensity due to COVID-19. Gerontol Geriatr Med.

[33] Reca I, Álvarez M, Tijoux M, de la Salud OP (2008). Costos no visibles del cuidado de enfermos en el hogar. Estudio de casos en Chile. La economía invisible y las desigualdades de género La importancia de medir y valorar el trabajo no remunerado.

[34] Jaracz K, Grabowska-Fudala B, Górna K, Jaracz J, Moczko J, Kozubski W (2015). Burden in caregivers of long-term stroke survivors: Prevalence and determinants at 6 months and 5 years after stroke. Patient Educ Couns.

[35] Oliva-Moreno J, Trapero-Bertran M, Peña-Longobardo LM, Del Pozo-Rubio R (2017). The valuation of informal care in cost-of-illness studies: a systematic review. Pharmacoeconomics.

[36] Zhu W, Jiang Y (2018). A meta-analytic study of predictors for informal caregiver burden in patients with stroke. J Stroke Cerebrovasc Dis.

[37] Stevens B, Pezzullo L, Verdian L, Tomlinson J, George A, Bacal F (2018). The economic burden of heart conditions in Brazil. Arq Bras Cardiol.

[38] Oliva-Moreno J, Peña-Longobardo LM, Mar J (2018). Determinants of informal care, burden, and risk of burnout in caregivers of stroke survivors: the CONOCES study. Stroke.

[39] Yabroff KR, Kim Y (2009). Time costs associated with informal caregiving for cancer survivors. Cancer.

[40] Kamal KM, Covvey JR, Dashputre A, Ghosh S, Shah S, Bhosle M, Zacker C (2017). A systematic review of the effect of cancer treatment on work productivity of patients and caregivers. J Manag Care Spec Pharm.

[41] Duimering A, Turner J, Chu K (2020). Informal caregiver quality of life in a palliative oncology population. Support Care Cancer.

[42] Souliotis K, Kousoulakou H, Hillas G, Tzanakis N, Toumbis M, Vassilakopoulos T (2017). The direct and indirect costs of managing chronic obstructive pulmonary disease in Greece. Int J Chron Obstruct Pulmon Dis.

[43] Puerto Pedraza HM (2015). Calidad de vida en cuidadores familiares de personas en tratamiento contra el cáncer. Rev Cuid.

[44] Wittenberg E, Prosser LA (2013). Disutility of illness for caregivers and families: a systematic review of the literature. Pharmacoeconomics.

[45] Wittenberg E, James LP, Prosser LA (2019). Spillover effects on caregivers’ and family members' utility: a systematic review of the literature. Pharmacoeconomics.

[46] Wooldridge JM. Introducción a la econometría: un enfoque moderno. Madrid: Thomson. 2006. 2a. ed., xxxi, 960 p. ISBN: 84-9732-268-1

[47] INEGI. Encuesta Nacional de Ocupación y Empleo. 2020. www.inegi.org.mx. https://www.inegi.org.mx/rnm/index.php/catalog/497. Accessed 15 Sep 2020.

[48] INEC. Encuesta Nacional de Hogares. In: Instituto Nacional de Estadísticas y Censos. 2019. https://www.inec.cr/encuestas/encuesta-nacional-de-hogares. Accessed Nov 2020.

[49] INDEC, Instituto Nacional de Estadística y Censos de la REPUBLICA ARGENTINA. Encuesta Permanente de Hogares. In: Instituto Nacional de Estadística y Censos. 2020. https://www.indec.gob.ar/indec/web/Institucional-Indec-BasesDeDatos. Accessed Jul 2020.

[50] INEI. ENAHO. In: Instituto Nacional de Estadística e Informática - INEI. 2020. https://www.datosabiertos.gob.pe/dataset/encuesta-nacional-de-hogares-enaho-2019-instituto-nacional-de-estad%C3%ADstica-e-inform%C3%A1tica-inei. Accessed Jun 2020.

[51] INEC. ENEMDU. In: Instituto Nacional de Estadística y Censos. 2019. https://www.ecuadorencifras.gob.ec/enemdu-2019/. Accessed Aug 2020.

[52] DANE. GEIH. In: Departamento Administrativo Nacional de Estadística (DANE). 2019. https://microdatos.dane.gov.co/index.php/catalog/599/datafile/F351/V18518. Accessed Feb 2020.

[53] Observatorio Social (2017–2018) CASEN. In: Observatorio Social. http://observatorio.ministeriodesarrollosocial.gob.cl/encuesta-casen. Accessed Oct 2019.

[54] IBGE. PNAD. In: Instituto Brasileiro de Geografía e Estadística. 2020. https://www.ibge.gov.br/en/statistics/social/labor/16833-monthly-dissemination-pnadc1.html?=&t=o-que-e. Accessed Nov 2020.

[55] www.bcra.gob.ar. In: Banco Central de la República Argentina. 2022. http://www.bcra.gob.ar/PublicacionesEstadisticas/Tipos_de_cambios.asp. Accessed 24 Feb 2022.

[56] www.bce.fin.ec. In: Banco Central de Ecuador. 2022. https://www.bce.fin.ec/index.php/component/k2/item/260-consulta-por-monedas-extranjeras. Accessed 24 Feb 2022.

[57] www.banxico.org.mx. In: Banco Central de México. 2022. https://www.banxico.org.mx/SieInternet/consultarDirectorioInternetAction.do?sector=6&accion=consultarCuadro&idCuadro=CF372&locale=es. Accessed 24 Feb 2022.

[58] totoro.banrep.gov.co. In: Banco Central de Colombia. 2022. https://totoro.banrep.gov.co/analytics/saw.dll?Go&NQUser=publico&NQPassword=publico123&Action=prompt&path=%2Fshared%2FSeries%20Estad%C3%ADsticas_T%2F1.%20Tasa%20de%20Cambio%20Peso%20Colombiano%2F1.1%20TRM%20-%20Disponible%20desde%20el%2027%20de%20noviembre%20de%201991%2F1.1.12.TCM_Serie%20historica%20promedio%20mensual&Options=rdf. Accessed 24 Feb 2022.

[59] www.bcentral.cl. In: Banco Central de Chile. 2022. https://www.bcentral.cl/web/banco-central/paridades-tipos-de-cambio-excel. Accessed 24 Feb 2022.

[60] estadisticas.bcrp.gob.pe. In: Banco Central de Reserva del Perú. 2022. https://estadisticas.bcrp.gob.pe/estadisticas/series/anuales/tipo-de-cambio-sol-usd. Accessed 24 Feb 2022.

[61] gee.bccr.fi.cr. In: Banco Central de Costa Rica. 2022. https://gee.bccr.fi.cr/indicadoreseconomicos/Cuadros/frmVerCatCuadro.aspx?CodCuadro=400&Idioma=1&FecInicial=2020/01/01&FecFinal=2020/12/31&Filtro=0. Accessed 24 Feb 2022.

[62] OECD. OECD Statistics. In: Organization for Economic Cooperation and Development (OECD). 2022. https://stats.oecd.org/. Accessed 29 Jan 2022.

[63] The World Bank. World Development Indicators. In: The World Bank. 2022. https://databank.worldbank.org/source/world-development-indicators. Accessed 29 Jan 2022.

[64] Pichon-Riviere A, Alcaraz A, Palacios A (2020). The health and economic burden of smoking in 12 Latin American countries and the potential effect of increasing tobacco taxes: an economic modelling study. Lancet Glob Health.

[65] Moerman CJ, van Mens-Verhulst J (2004). Gender-sensitive epidemiological research: suggestions for a gender-sensitive approach towards problem definition, data collection and analysis in epidemiological research. Psychol Health Med.

[66] Nowatzki N, Grant KR (2011). Sex is not enough: the need for gender-based analysis in health research. Health Care Women Int.

[67] Pederson A, Greaves L, Poole N (2015). Gender-transformative health promotion for women: a framework for action. Health Promot Int.

[68] Williams CR, Bennett E, Meier BM. Incorporating a gender perspective to realise the health and human rights of older persons. ageing, health and international law: towards an international legal framework to advance the health and human rights of older persons (Allyn Taylor & Patricia Kuszler, eds). 2020. 10.2139/ssrn.3790125.

