# Supplementary material for: Making visible the cost of informal caregivers’ time in Latin America: a case study for major cardiovascular, cancer and respiratory diseases in eight countries
Source: BMC Public Health. 2023 Jan 5;23:28. doi: 10.1186/s12889-022-14835-w (PMC9815678; doi:10.1186/s12889-022-14835-w)
Supplement: Supplementary file 1 — Additional file 1: SM. Search strategies in the literature review. Table S1. Inputs used to estimate the hours per day of informal care by events. Table S2. Hourly wage used to proxy of the market value of informal care time, rate exchange and GDP by country, 2020 USD. Table S3. Total number of cases per health events and country in a year. Table S4. Medical direct costs per health event and country, million 2020 USD. Table S5. Average cost per day of informal care by health condition and country, in 2020 dollars. [file 12889_2022_14835_MOESM1_ESM.docx]

**Appendix**

[Supplementary information 2](#_Toc113648851)

[Search strategies in the literature review 2](#_Toc113648852)

[Supplementary Table 4](#_Toc113648853)

[Table S1. Inputs used to estimate the hours per day of informal care by events 4](#_Toc113648854)

[Table S2. Hourly wage used to proxy of the market value of informal care time, rate exchange and GDP by country, 2020 USD 6](#_Toc113648855)

[Table S3. Total number of cases per health events and country in a year 6](#_Toc113648856)

[Table S4. Medical direct costs per health event and country, million 2020 USD 7](#_Toc113648857)

[Table S5. Average cost per day of informal care by health condition and country, in 2020 dollars 8](#_Toc113648858)

# **Supplementary information**

## **Search strategies in the literature review**

**PUBMED**

Cardiovascular diseases

(Caregivers[Mesh] OR Care Giver*[tiab] OR Caregiver*[tiab] OR Informal Care*[tiab]) AND (Cardiovascular Diseases[Mesh] OR Cardiovascular[tiab] OR Myocardial Ischemia[Mesh] OR Acute Coronar*[tiab] OR Coronary Syndrom*[tiab] OR STEMI[tiab] OR non-STEMI[tiab] OR Myocardial isch*[tiab] OR Stroke*[tiab]) AND (Time Management[Mesh] OR Time and Motion Studies[Mesh] OR Use-of-Time[tiab] OR time[tiab] OR hours[tiab] OR Economic burden[tiab] OR burden[tiab] OR Disease Cost*[tiab] OR Indirect Cost*[tiab] OR cost-of-illness[tiab]) AND (Systematic Review[sb] OR Systematic Review[tiab] OR Meta-Analysis[pt] OR Meta-Analys*[tiab] OR "Cochrane Database Syst Rev"[ta] OR Metaanalysis[tiab] OR Metanalysis[tiab] OR Overview[ti] OR (Review[ti] AND Literature[ti]) OR (MEDLINE[tiab] AND Cochrane[tiab]))

Cancers

(Caregivers[Mesh] OR Care Giver*[tiab] OR Caregiver*[tiab] OR Informal Care*[tiab]) AND (Neoplasms[Mesh] OR Cancer[tiab]) AND (Time Management[Mesh] OR Time and Motion Studies[Mesh] OR Use-of-Time[tiab] OR time[tiab] OR hours[tiab] OR Economic burden[tiab] OR burden[tiab] OR Disease Cost*[tiab] OR Indirect Cost*[tiab] OR cost-of-illness[tiab]) AND (Systematic Review[sb] OR Systematic Review[tiab] OR Meta-Analysis[pt] OR Meta-Analys*[tiab] OR "Cochrane Database Syst Rev"[ta] OR Metaanalysis[tiab] OR Metanalysis[tiab] OR Overview[ti] OR (Review[ti] AND Literature[ti]) OR (MEDLINE[tiab] AND Cochrane[tiab]))

Pulmonary diseases.

(Caregivers[Mesh] OR Care Giver*[tiab] OR Caregiver*[tiab] OR Informal Care*[tiab]) AND (Pulmonary Disease, Chronic Obstructive[Mesh] OR COPD[tiab] OR Chronic Obstructive[tiab] OR Obstructive Airway[tiab] OR Pulmonary Disease*[tiab]) AND (Time Management[Mesh] OR Time and Motion Studies[Mesh] OR Use-of-Time[tiab] OR time[tiab] OR hours[tiab] OR Economic burden[tiab] OR burden[tiab] OR Disease Cost*[tiab] OR Indirect Cost*[tiab] OR cost-of-illness[tiab]) AND (Systematic Review[sb] OR Systematic Review[tiab] OR Meta-Analysis[pt] OR Meta-Analys*[tiab] OR "Cochrane Database Syst Rev"[ta] OR Metaanalysis[tiab] OR Metanalysis[tiab] OR Overview[ti] OR (Review[ti] AND Literature[ti]) OR (MEDLINE[tiab] AND Cochrane[tiab]))

**LILACS**

(MH Caregivers OR Caregiver$ OR Cuidador$ OR Carer) AND (Tiempo OR Tempo OR Time) AND (MH Neoplasms OR MH Pneumonia OR MH Cardiovascular Diseases OR Cardiovascular OR Cancer OR Coronar$ OR EPOC OR stroke OR ACV OR Cerebrovascular OR Chronic obstructive pulmonary disease)

# **Supplementary Table**

## **Table S1. Inputs used to estimate the hours per day of informal care by events**

| **Events** | **Utility** | **Source** | **Care days per year[15]** | **Use rate[15]** |
| --- | --- | --- | --- | --- |
| **Acute Myocardial Infarction (first year)** | 0.803 | Smith [1] | 30 | 65% |
| **Coronary event no AMI (first year)** | 0.803 | Smith [1] | 30 | 65% |
| **Coronary event (year 2+)** | 0.850 | Wijeysundera [2] | 365 | 5% |
| **Stroke (first year)** | 0.620 | Yeoh [3] | 60 | 33% |
| **Stroke (year 2+)** | 0.780 | Yeoh [3] | 365 | 17% |
| **Mild COPD** | 0.935 | Mölken [4] | 365 | 0% |
| **Moderate COPD** | 0.776 | Mölken[4] | 365 | 60% |
| **Severe COPD** | 0.689 | Mölken [4] | 365 | 80% |
| **Pneumonia** | 0.994 | Pepper [5] | 30 | 65% |
| **Lung cancer** | 0.660 | Chouaid [6] | 365 | 100% |
| **Mouth cancer** | 0.745 | Nie [7] | 365 | 100% |
| **Esophageal cancer** | 0.630 | Graham [8] | 365 | 100% |
| **Stomach cancer** | 0.550 | Dan [9] | 365 | 100% |
| **Pancreatics cancer** | 0.550 | Gordois [10] | 365 | 100% |
| **Kidney cancer** | 0.780 | Pickard [11] | 365 | 100% |
| **Laryngeal cancer** | 0.760 | Pickard [11] | 365 | 100% |
| **Leukemia** | 0.820 | Leunis [12] | 365 | 100% |
| **Bladder cancer** | 0.678 | Hevér [13] | 365 | 100% |
| **Neck cancer** | 0.758 | Endarti [14] | 365 | 100% |

Note:

Equation resulting from the econometric estimation for missing data of informal care hours of time: y= -13.32*x + 12.325

References:

1 Smith DW, Davies EW, Wissinger E, Huelin R, Matza LS, Chung K. A systematic literature review of cardiovascular event utilities. Vol. 13, Expert Review of Pharmacoeconomics and Outcomes Research. 2013.

2 Wijeysundera HC, Farshchi-Zarabi S, Witteman W, Bennell MC. Conversion of the Seattle Angina questionnaire into EQ-5D utilities for ischemic heart disease: A systematic review and catalog of the literature. Clin Outcomes Res. 2014;6(1).

3 Yeoh YS, Koh GCH, Tan CS, Tu TM, Singh R, Chang HM, et al. Health-related quality of life loss associated with first-time stroke. PLoS One. 2019;14(1).

4 Mölken MR Van, Lee TA. Economic modeling in chronic obstructive pulmonary disease. In: Proceedings of the American Thoracic Society. 2006.

5 Pepper PV, Owens DK. Cost-effectiveness of the pneumococcal vaccine in healthy younger adults. Med Decis Mak. 2002;22(5 SUPPL.).

6 Chouaid C, Agulnik J, Goker E, Herder GJM, Lester JF, Vansteenkiste J, et al. Health-related quality of life and utility in patients with advanced non-small-cell lung cancer: A prospective cross-sectional patient survey in a real-world setting. J Thorac Oncol. 2013;8(8).

7 Nie M, Liu C, Pan YC, Jiang CX, Li BR, Yu XJ, et al. Development and evaluation of oral Cancer quality-of-life questionnaire (QOL-OC). BMC Cancer. 2018;18(1).

8 Graham AJ, Shrive FM, Ghali WA, Manns BJ, Grondin SC, Finley RJ, et al. Defining the Optimal Treatment of Locally Advanced Esophageal Cancer: A Systematic Review and Decision Analysis. Ann Thorac Surg. 2007;83(4).

9 Dan YY, So JBY, Yeoh KG. Endoscopic Screening for Gastric Cancer. Clin Gastroenterol Hepatol. 2006;4(6).

10 Gordois A, Scuffham P, Warren E, Ward S. Cost-utility analysis of imatinib mesilate for the treatment of advanced stage chronic myeloid leukaemia. Br J Cancer. 2003;89(4).

11 Pickard AS, Jiang R, Lin HW, Rosenbloom S, Cella D. Using Patient-reported Outcomes to Compare Relative Burden of Cancer: EQ-5D and Functional Assessment of Cancer Therapy-General in Eleven Types of Cancer. Clin Ther. 2016;38(4).

12 Leunis A, Redekop WK, Uyl-de Groot CA, Löwenberg B. Impaired health-related quality of life in acute myeloid leukemia survivors: A single-center study. Eur J Haematol. 2014;93(3).

13 Hevér N V., Péntek M, Balló A, Gulácsi L, Baji P, Brodszky V, et al. Health Related Quality of Life in Patients with Bladder Cancer: A Cross-Sectional Survey and Validation Study of the Hungarian Version of the Bladder Cancer Index. Pathol Oncol Res. 2015;21(3).

14 Endarti D, Riewpaiboon A, Thavorncharoensap M, Praditsitthikorn N, Hutubessy R, Kristina SA. Evaluation of health-related quality of life among patients with cervical cancer in Indonesia. Asian Pacific J Cancer Prev. 2015;16(8).

15 Own elaboration based Interviews with health professionals

## **Table S2. Hourly wage used to proxy of the market value of informal care time, rate exchange and GDP by country, 2020 USD**

| **Countries** | **Wage per hour** | | | **Rate exchange (LC/USD)** | **GDP (in million USD)** |
| --- | --- | --- | --- | --- | --- |
|  | **Mean** | **IC 95%** | |  |  |
|  |  | **IL** | **UL** |  |  |
| Argentina | 2.32 | 1.24 | 2.85 | 70.63 | $421237.14 |
| Brazil | 2.59 | 1.10 | 2.76 | 5.38 | $1273118.68 |
| Chile | 4.12 | 2.38 | 4.50 | 792.17 | $240312.29 |
| Colombia | 1.34 | 1.25 | 2.24 | 3693.28 | $270842.50 |
| Costa Rica | 2.91 | 2.81 | 3.62 | 584.94 | $59698.45 |
| Ecuador | 2.97 | 2.51 | 3.39 | 1 | $100667.53 |
| Mexico | 1.05 | 0.18 | 1.49 | 21.5 | $1051525.22 |
| Peru | 2.39 | 1.39 | 2.15 | 3.49 | $207161.12 |

Source: Own elaboration based national surveys.

Note: LC: Local currency

## **Table S3. Total number of cases per health events and country in a year**

| **Events** | **Argentina** | **Brazil** | **Chile** | **Colombia** | **Costa Rica** | **Ecuador** | **Peru** | **Mexico** |
| --- | --- | --- | --- | --- | --- | --- | --- | --- |
| **Acute Myocardial Infarction (first year)** | 107539 | 877966 | 42998 | 181679 | 7627 | 59770 | 20716 | 387114 |
| **Coronary event no AMI (first year)** | 169148 | 1033378 | 67972 | 156007 | 33503 | 16085 | 12902 | 250370 |
| **Coronary event (year 2+)** | 2301017 | 17315235 | 1024722 | 1900212 | 334026 | 638821 | 318016 | 4660273 |
| **Stroke (first year)** | 94631 | 345054 | 74802 | 183357 | 5683 | 37924 | 53016 | 261026 |
| **Stroke (year 2+)** | 664481 | 2680302 | 522322 | 1432941 | 30919 | 259314 | 428598 | 1887680 |
| **Pneumonia** | 183185 | 792625 | 29072 | 57286 | 7178 | 34239 | 110662 | 265385 |
| **Mild COPD** | 1719609 | 7143987 | 966812 | 1569348 | 132836 | 400352 | 1203821 | 3305023 |
| **Moderate COPD** | 373735 | 1522848 | 215242 | 346907 | 27934 | 84071 | 275575 | 707501 |
| **Severe COPD** | 72284 | 288830 | 42455 | 68290 | 5262 | 15780 | 57045 | 135897 |
| **Lung cancer** | 34329 | 90045 | 12685 | 15910 | 1443 | 2859 | 9085 | 24693 |
| **Mouth cancer** | 9689 | 62758 | 2644 | 6629 | 713 | 1166 | 4341 | 11362 |
| **Esophageal cancer** | 9460 | 42560 | 3491 | 3584 | 371 | 744 | 1675 | 4798 |
| **Stomach cancer** | 16301 | 76348 | 19268 | 28058 | 3531 | 8540 | 21363 | 28943 |
| **Pancreatics cancer** | 21101 | 53060 | 7804 | 10399 | 1185 | 2164 | 6571 | 19681 |
| **Kidney cancer** | 18041 | 29995 | 8375 | 5101 | 710 | 1425 | 5519 | 16665 |
| **Laryngeal cancer** | 6395 | 34078 | 1351 | 3311 | 453 | 564 | 1041 | 5793 |
| **Leukemia** | 10461 | 35677 | 4993 | 9160 | 974 | 2729 | 6334 | 15315 |
| **Bladder cancer** | 15049 | 45839 | 5893 | 5941 | 831 | 1291 | 3309 | 7841 |
| **Neck cancer** | 15193 | 51752 | 5770 | 13069 | 1128 | 4898 | 13883 | 25979 |
| **Total** | 5841647 | 32522338 | 3058670 | 5997190 | 596306 | 1572736 | 2553472 | 12021339 |

Source: Extracted and updated to Pichon-Riviere et al (2020)

## **Table S4. Medical direct costs per health event and country, million 2020 USD**

| Events | Argentina | Brazil | Chile | Colombia | Costa Rica | Ecuador | Mexico | Peru |
| --- | --- | --- | --- | --- | --- | --- | --- | --- |
| Coronary events | 4059 | 10195 | 1877 | 2817 | 693 | 1286 | 8232 | 493 |
| Stroke | 405 | 2720 | 1139 | 794 | 89 | 430 | 2223 | 655 |
| COPD | 744 | 6090 | 628 | 489 | 86 | 241 | 2775 | 567 |
| Pneumonia | 93 | 215 | 7 | 41 | 13 | 16 | 335 | 21 |
| Cancers | 2375 | 2753 | 1213 | 684 | 239 | 322 | 1493 | 855 |
| Total | 7676 | 21973 | 4863 | 4824 | 1120 | 2295 | 15058 | 2590 |

Source: Extracted and updated to Pichon-Riviere et al (2020)

## **Table S5. Average cost per day of informal care by health condition and country, in 2020 dollars**

| **Events** | **Argentina** | | **Brazil** | | **Chile** | | **Colombia** | | **Costa Rica** | | **Ecuador** | | **Peru** | | **Mexico** | |
| --- | --- | --- | --- | --- | --- | --- | --- | --- | --- | --- | --- | --- | --- | --- | --- | --- |
|  | Mean | IC 95% | Mean | IC 95% | Mean | IC 95% | Mean | IC 95% | Mean | IC 95% | Mean | IC 95% | Mean | IC 95% | Mean | IC 95% |
| **Acute Myocardial Infarction (first year)** | 2.38 | [2.06-2.69] | 2.65 | [1.97-3.54] | 4.22 | [3.67-4.78] | 1.37 | [1.1-1.71] | 2.98 | [2.61-3.37] | 3.05 | [1.43-6.15] | 2.45 | [1.17-4.78] | 1.07 | [0.94-1.24] |
| **Coronary event no AMI (first year)** | 2.24 | [1.87-2.64] | 2.49 | [1.81-3.49] | 3.97 | [3.32-4.66] | 1.29 | [1.02-1.67] | 2.81 | [2.37-3.33] | 2.87 | [1.3-5.83] | 2.30 | [1.04-4.57] | 1.01 | [0.86-1.19] |
| **Coronary event (post event)** | 1.16 | [0.91-1.46] | 1.30 | [0.9-1.86] | 2.06 | [1.61-2.61] | 0.67 | [0.5-0.9] | 1.46 | [1.14-1.84] | 1.49 | [0.69-3.12] | 1.20 | [0.54-2.43] | 0.52 | [0.41-0.66] |
| **Stroke (first year)** | 3.53 | [3.23-3.85] | 3.94 | [3.01-5.29] | 6.26 | [5.69-6.9] | 2.04 | [1.69-2.5] | 4.43 | [4.05-4.82] | 4.53 | [2.09-9.31] | 3.64 | [1.71-7.42] | 1.59 | [1.47-1.74] |
| **Stroke (year 2+)** | 2.41 | [2.08-2.78] | 2.69 | [2.05-3.69] | 4.28 | [3.68-4.95] | 1.40 | [1.12-1.76] | 3.03 | [2.63-3.5] | 3.10 | [1.4-6.46] | 2.49 | [1.15-4.96] | 1.09 | [0.95-1.25] |
| **Pneumonia** | 0.83 | [0.5-1.36] | 0.93 | [0.53-1.64] | 1.48 | [0.89-2.39] | 0.48 | [0.28-0.81] | 1.05 | [0.64-1.68] | 1.07 | [0.46-2.52] | 0.86 | [0.36-1.97] | 0.38 | [0.24-0.61] |
| **Mild COPD** | 0.00 | - | 0.00 | - | 0.00 | - | 0.00 | - | 0.00 | - | 0.00 | - | 0.00 | - | 0.00 | - |
| **Moderate COPD** | 1.59 | [1.25-2.06] | 1.78 | [1.28-2.65] | 2.83 | [2.19-3.67] | 0.92 | [0.68-1.25] | 2.00 | [1.57-2.58] | 2.04 | [0.93-4.33] | 1.64 | [0.76-3.36] | 0.72 | [0.56-0.94] |
| **Severe COPD** | 13.27 | [12.83-13.7] | 14.81 | [11.37-19.38] | 23.55 | [22.37-24.81] | 7.67 | [6.44-9.24] | 16.66 | [16.2-17.08] | 17.03 | [8.07-34.76] | 13.67 | [6.61-27.13] | 5.98 | [5.83-6.16] |
| **Lung cancer** | 8.21 | [7.83-8.56] | 9.16 | [7.06-12.21] | 14.57 | [13.67-15.5] | 4.74 | [3.95-5.71] | 10.30 | [9.88-10.69] | 10.53 | [4.93-21.05] | 8.45 | [4.11-16.53] | 3.70 | [3.56-3.88] |
| **Mouth cancer** | 5.58 | [5.26-5.89] | 6.22 | [4.82-8.28] | 9.90 | [9.2-10.62] | 3.22 | [2.68-3.9] | 7.00 | [6.61-7.4] | 7.16 | [3.3-14.32] | 5.75 | [2.75-11.46] | 2.51 | [2.38-2.66] |
| **Esophageal cancer** | 9.13 | [8.74-9.52] | 10.19 | [7.86-13.45] | 16.21 | [15.23-17.29] | 5.28 | [4.44-6.36] | 11.46 | [11.05-11.89] | 11.72 | [5.49-23.73] | 9.41 | [4.59-18.67] | 4.12 | [3.98-4.29] |
| **Stomach cancer** | 11.61 | [11.23-12] | 12.95 | [10-17.08] | 20.60 | [19.45-21.76] | 6.71 | [5.6-8.07] | 14.57 | [14.18-14.95] | 14.90 | [7.03-30.04] | 11.96 | [5.8-23.77] | 5.23 | [5.09-5.4] |
| **Pancreatics cancer** | 11.61 | [11.25-11.98] | 12.95 | [10.03-17.05] | 20.60 | [19.52-21.75] | 6.71 | [5.6-8.05] | 14.57 | [14.19-14.95] | 14.90 | [6.97-30.19] | 11.96 | [5.82-23.59] | 5.23 | [5.09-5.4] |
| **Kidney cancer** | 4.49 | [4.19-4.85] | 5.01 | [3.89-6.61] | 7.98 | [7.34-8.76] | 2.60 | [2.14-3.2] | 5.64 | [5.25-6.06] | 5.77 | [2.65-12] | 4.63 | [2.21-9.11] | 2.03 | [1.89-2.19] |
| **Laryngeal cancer** | 5.11 | [4.76-5.47] | 5.71 | [4.42-7.58] | 9.08 | [8.37-9.88] | 2.96 | [2.46-3.62] | 6.42 | [6.01-6.85] | 6.56 | [3.06-13.5] | 5.27 | [2.46-10.37] | 2.31 | [2.15-2.47] |
| **Leukemia** | 3.26 | [2.97-3.57] | 3.63 | [2.76-4.87] | 5.78 | [5.24-6.4] | 1.88 | [1.56-2.29] | 4.09 | [3.74-4.47] | 4.18 | [1.97-8.38] | 3.36 | [1.57-6.62] | 1.47 | [1.34-1.62] |
| **Bladder cancer** | 7.65 | [7.29-7.98] | 8.54 | [6.59-11.2] | 13.58 | [12.77-14.4] | 4.42 | [3.7-5.3] | 9.60 | [9.19-9.96] | 9.82 | [4.6-19.85] | 7.88 | [3.75-15.47] | 3.45 | [3.31-3.59] |
| **Neck cáncer** | 5.17 | [4.84-5.54] | 5.77 | [4.4-7.66] | 9.19 | [8.46-9.99] | 2.99 | [2.49-3.64] | 6.50 | [6.09-6.94] | 6.64 | [3.09-13.71] | 5.33 | [2.54-10.37] | 2.33 | [2.19-2.49] |
